# A Suite of Therapeutically-Inspired Nucleic Acid Logic Systems for Conditional Generation of Single-Stranded and Double-Stranded Oligonucleotides

**DOI:** 10.3390/nano9040615

**Published:** 2019-04-15

**Authors:** Paul Zakrevsky, Eckart Bindewald, Hadley Humbertson, Mathias Viard, Nomongo Dorjsuren, Bruce A. Shapiro

**Affiliations:** 1 RNA Biology Laboratory, National Cancer Institute, Frederick, MD 21702, USA; paul.zakrevsky@nih.gov (P.Z.); hadley.humbertson@nih.gov (H.H.); nomiko.d@gmail.com (N.D.); 2 Basic Science Program, Frederick National Laboratory for Cancer Research, Frederick, MD 21702, USA; eckart@mail.nih.gov (E.B.); mathias.viard@nih.gov (M.V.)

**Keywords:** RNA, RNA logic, conditional activation, functional RNA, nucleic acid therapeutic

## Abstract

Several varieties of small nucleic acid constructs are able to modulate gene expression via one of a number of different pathways and mechanisms. These constructs can be synthesized, assembled and delivered to cells where they are able to impart regulatory functions, presenting a potential avenue for the development of nucleic acid-based therapeutics. However, distinguishing aberrant cells in need of therapeutic treatment and limiting the activity of deliverable nucleic acid constructs to these specific cells remains a challenge. Here, we designed and characterized a collection of nucleic acids systems able to generate and/or release sequence-specific oligonucleotide constructs in a conditional manner based on the presence or absence of specific RNA trigger molecules. The conditional function of these systems utilizes the implementation of *AND* and *NOT* Boolean logic elements, which could ultimately be used to restrict the release of functionally relevant nucleic acid constructs to specific cellular environments defined by the high or low expression of particular RNA biomarkers. Each system is generalizable and designed with future therapeutic development in mind. Every construct assembles through nuclease-resistant RNA/DNA hybrid duplex formation, removing the need for additional 2′-modifications, while none contain any sequence restrictions on what can define the diagnostic trigger sequence or the functional oligonucleotide output.

## 1. Introduction

Deliverable nucleic acid-based systems present powerful methods to modulate specific gene expression and have the potential to be developed for therapeutic purposes, however, restricting their activity to a subset of intended cells remains challenging [1,2,3]. Numerous methods that utilize relatively small synthetic nucleic acids to regulate endogenous gene expression have originated in recent years, providing several approaches for which targeted therapeutics can be developed. The use of single-stranded antisense oligonucleotides (AON) were among the first of these regulatory techniques, whereby an oligonucleotide complementary to an mRNA of interest could be used to regulate expression of that gene [4]. AONs were originally designed to inhibit gene expression through steric inhibition of the translation machinery but have evolved into several parallel regulatory approaches. These include targeted degradation of mRNA through RNaseH activity [5], as well as alteration of mRNA splicing patterns by limiting the accessibility of specific splice sites [6]. RNA interference (RNAi) methods then followed, again evolving from the general notion of double stranded RNA being able to silence a complementary target mRNA [7], to the design of synthetic short interfering RNA (siRNA) [8], Dicer substrate siRNA (DsiRNA) [9], and other related constructs [10,11]. More recently, analogous approaches have been developed that increase rather than downregulate expression of a target gene. Delivery of double-stranded siRNA-like duplexes termed short activating RNA (saRNA) has been observed to activate gene expression when targeted to promotor regions [12], while single-stranded antagomiRs (also referred to as antimiRs) can be designed to sequester mature endogenous microRNAs and inhibit their native regulatory function [13]. Despite the development of diverse regulatory approaches, the prospect of these functional nucleic acids altering gene expression in non-afflicted healthy cells can be a cause for concern and presents a roadblock towards clinical development.

Strategies to date for cell-specific functions of nucleic acid constructs can largely be divided into two categories: targeted delivery and conditional activation. Targeted delivery most often involves the conjugation of a functionally active nucleic acid to a small molecule, protein, aptamer or other targeting agent that interacts with a receptor specifically expressed on the target cells of interest [14,15,16,17]. This approach can be effective but requires the development or identification of a ligand for the particular target cells of interest, the conjugation of such a ligand to the functional nucleic acid, and necessitates that the ligand/receptor interaction induces internalization of the nucleic acid payload.

Conditional activation represents an opposite approach to targeted delivery, in which the functional nucleic acid could be systemically delivered in an inactive state and only performs its active function in a subset of specified cells. This can be achieved through the implementation of nucleic acid logic elements that promote generation of a functional oligonucleotide construct through recognition of a specific cellular environment. The occurrence of disease can often be the result of, as well as result in, the mis-regulation of gene expression, and these differentially expressed genes can be used as biomarkers to distinguish corrupted cells from healthy tissue [18,19,20]. Numerous systems have been devised that incorporate nucleic acid logic elements to perform a specified function or generate predetermined molecular outputs conditional on the presence of an input oligonucleotide sequence. These include systems such as molecular beacons [21,22] and allosteric ribozymes [23,24], as well as nucleic acid strand exchange events and strand displacement cascades [25,26,27,28,29,30,31,32]. Several incarnations of nucleic acid logic systems have been previously devised which are able to release an oligonucleotide product in a conditional manner. For such approaches to be amenable to practical therapeutic application and systemic delivery, they should ideally be robust in their design to accommodate great diversity in terms of input and output oligonucleotide sequences, protect their RNA components from ribonuclease degradation, and be cheap and efficient to synthesize and produce. Existing strand exchange systems often fulfill one or two of these requirements, but rarely meet these criteria in their entirety.

Here, we present the design of several new systems for the conditional release of single-stranded (ss) and double-stranded (ds) nucleic acid constructs that are specifically tailored to meet these criteria of ideal characteristics, which many existing systems fail to adequately satisfy. These designs are influenced by several existing nucleic acid technologies such as cognate RNA/DNA hybrids, molecular beacons, and trigger-responsive multi-stranded switch constructs [21,31,32,33,34], with the aim to take the most favorable characteristics from existing systems and create derivative systems with improved features [35]. Within each construct, the diagnostic region is structurally separated from the oligonucleotide payload, resulting in systems where input and output sequences are completely decoupled and impart no sequence constraints on one another. Additionally, any RNA strands are initially bound within RNA/DNA hybrid duplexes to provide resistance from ribonuclease degradation without the need of additional 2′-modifications [31], as these modifications can increase the costs and reduce efficiency of commercial oligonucleotide synthesis. Furthermore, we expand the degree of conditional control commonly observed in systems designed for conditional generation of sequence specific dsRNA by demonstrating that conditional dsRNA release can not only be induced but also repressed upon interaction with an RNA trigger, culminating in a cognate pair of RNA/DNA hybrid constructs for which dsRNA release is under the control of multiple input triggers. As a complete collection, these novel conditional systems provide an assortment of diversity in terms of addressing different scenarios for treatment (ss vs. ds oligo release) and diagnosis (biomarker mediated induction or repression). Ultimately, this assemblage of conditional nucleic acid systems can be modified to harbor components with biologically relevant function and developed to act as conditionally regulated therapeutics.

## 2. Materials and Methods 

### 2.1. Computational Considerations and RNA/DNA Hybrid Construct Design

The computational folding of individual strands and assembly of DNA/RNA constructs was assessed using Hyperfold [32], a nucleic acid structure prediction algorithm capable of predicting multi-strand assemblies from combinations of RNA and DNA strands. All folding predictions were performed at strand concentrations of 1 µM at 37 °C. The visualization and depiction of resulting secondary structure predictions was performed using Ribosketch [36]. A detailed design description of RNA/DNA hybrid pairs can be found in supporting information.

### 2.2. Oligonucleotide Synthesis and Purification

The DNA and RNA oligonucleotides used to assemble the conditional RNA/DNA constructs, including those that were fluorescently labeled, were purchased from Integrated DNA Technologies (IDT, Coralville, IA, USA) and reconstituted in nuclease-free water (Quality Biological, Gaithersburg, MD, USA) for use. All AlexaFluor546, AlexaFluor488 and 6-carboxyfluorescein (6-FAM) fluorescently labeled oligonucleotides were purchased from IDT. For commercially purchased oligonucleotides, 10 nmol quantities were purified as needed by denaturing polyacrylamide gel electrophoresis (PAGE). Specifically, 10 nmol quantities were mixed with 100 uL urea loading buffer (6 M urea, 20 mM ethylenediaminetetraacetic acid (EDTA), 10% glycerol, 0.05% bromophenol blue) and heated to 90 °C for 2 min prior to loading on an 8% or 10% 19:1 acrylamide/bis-acrylamide denaturing gel (1× TBE buffer (89 mM Tris, 89 mM boric acid, 2 mM EDTA), 6 M Urea) for purification. Following electrophoresis, bands were cut from the gel and eluted in an elution buffer (10 mM Tris pH 7.5, 200 mM NaCl, 0.5 mM EDTA) overnight at 4 °C while shaken at 850 rpm. Eluted oligonucleotides were ethanol precipitated and reconstituted in nuclease-free water.

RNA trigger oligonucleotides either purchased from IDT or prepared from in vitro runoff transcription using T7 RNA polymerase. DNA templates for transcription were amplified by PCR using primers purchased form IDT. PCR was performed using MyTaq 2× mix (Bioline, Memphis, TN, USA) and purified using DNA Clean & Concentrator (Zymo Research, Irvine, CA, USA). Transcription was performed in 10 mM Tris pH 7.0 containing 6 mM MgCl_2_, 0.5 mM MnCl_2_, 2.5 mM each NTP, 0.01 u/µL inorganic pyrophosphatase, 2 mM dithiothreitol, and 2 mM spermidine. Approximately 50 pmol of DNA template was added to the transcription mix along with an in-house produced T7 RNA polymerase and incubated at 37 °C for 4 h. Transcription was terminated by addition of DNase I (New England Biolabs, Ipswich, MA, USA) for 30 min. The transcription mix was combined with 1/2 volume of urea loading buffer and heated at 90 °C for 2 min before purification by denaturing PAGE and precipitation as described above.

### 2.3. RNA/DNA Construct Assembly

Conditional RNA/DNA constructs were assembled using equimolar concentrations of their component strands. Strands were combined in water, heated to 90 °C for 1.5 min, and then immediately placed on a 37 °C heat block for 5 min. After this, samples were briefly spun in a tabletop centrifuge to collect condensed solvent and assembly buffer was added to a final 1× concentration of 2 mM Mg(OAc)_2_, 50 mM KCl, 1× TB (89 mM Tirs, 89 mM boric acid, pH 8.2). The assembly was then incubated an additional 25 min at 37 °C. Control dsRNA duplexes and RNA trigger molecules were assembled/folded using the same protocol.

### 2.4. Non-Denaturing PAGE Analysis of Conditional Oligonucleotide Release

Assembled constructs were examined for their ability to regulate conditional oligonucleotide release in the presence and absence of specific RNA trigger molecules. All constructs and triggers were initially prepared separately in 1× assembly buffer. From these bulk individual assemblies, various construct/trigger combinations were combined and incubated at 37 °C for either 30, 90 or 180 minutes. Individual controls were prepared from the same bulk assemblies and subjected to identical incubation conditions. Generally, the conditional constructs were present at a final concentration of 500 nM. In the case of the beacon switch and adjacent targeting hybrids, RNA triggers were present in a 1× concentration relative to the conditional constructs. For inducible and repressible hybrid systems, the RNA triggers were generally present at 2×–3× concentrations, as indicated in the text. Following this incubation, samples were transferred to ice, combined with 1/5 volume of loading buffer (1× assembly buffer, 50% glycerol) and were loaded on non-denaturing PAGE gels (8–12% 19:1 acrylamide/bis-acrylamide, 2 mM Mg(OAc)_2_, 1× TB). Electrophoresis was generally performed at 6 W for 2–3 h at 10 °C. Acrylamide concentrations and duration of electrophoresis were optimized on a case-by-case basis to achieve the necessary separation of species. In some instances, gels were subjected to total nucleic acid staining with ethidium bromide. In other instances, an individual molecule within a construct was fluorescently labeled (~10% of total molecules used in an assembly). In both cases, gels were imaged using a Typhoon Trio variable mode imager (GE Healthcare, Chicago, IL, USA) using appropriate excitation and emission filters. The amount of fluorescently labeled dsRNA output released from conditional systems was quantitated using ImageQuant 5.1 (Molecular Dynamics (now GE Healthcare), Chicago, IL, USA). Unless otherwise noted, the fraction of dsRNA released for a given sample is reported as the ratio of fluorescence observed in the released dsRNA band to the total amount of fluorescence observed for the entire lane. Statistical significance between populations was determined by a two-tailed Student’s *t*-test performed using values from three distinct replicate experiments.

### 2.5. Analysis of RNA/DNA Strand Exchange by Förster Resonance Energy Transfer (FRET)

RNA/DNA strand exchange between cognate partners of inducible and repressible hybrid systems were examined by FRET. Cognate hybrids were assembled separately, and pre-warmed to 37 °C. Hybrids were combined and added to the cuvette, at which point the RNA trigger molecule was spiked in, if appropriate. The cuvette was immediately placed in a FluoroMax-3 fluorimeter (Jobin Yvon Horiba, Kyoto, Japan) at 37 °C and measurement was started. For FRET experiments where a fluorescence spectrum was measured at a given time point, the sense hybrid was assembled with an RNA sense strand containing a 3′ 6-FAM donor fluorophore, while the antisense hybrid was assembled with an RNA antisense strand possessing a 5′ AlexaFluor546 acceptor fluorophore. Hybrids were prepared to a final concentration of 250 mM and the trigger molecule was in three-fold molar excess, when present. Excitation was performed at 475 nm and emission measured between 480–620 nm at 1 nm increments using 0.5 s integration times and 2 nm slit widths. 

For FRET experiments where time courses were recorded, the 6-FAM donor fluorophore on the RNA sense strand was replaced with AlexaFluor488. Hybrid and trigger concentration mirrored the conditions of analogous non-denaturing PAGE experiments, with hybrids at a final concentration of 500 nM, and trigger concentrations in 2–3 fold molar excess, as indicated. Measurements were recorded every 60 s using excitation at 475 nm and emission was measured at 515 nm and 565 nm, using a signal integration time of 0.5 s and slit widths of 2 nm. Observed rate constants (k_obs_) were obtained by fitting the decrease in measured AlexaFluor488 donor fluorescence as a function of time to the equation y = y_0_ + A*e*^−k*t^ for single exponential decay.

## 3. Results

### 3.1. Crafting a Collection of Diverse, Logic-Based Nucleic Acid Systems Geared Towards Therapeutic Development

Multiple nucleic acid systems were designed to specifically adhere to the ideal structural and functional criteria for an RNA-based conditional therapeutic that were outlined above. Each individual assembly is composed of only RNA and DNA oligonucleotides, with no additional 2′-modified nucleotides. Every initial-state assembly was designed to contain RNA/DNA hybrid duplexes to minimize potential ribonuclease cleavage of the RNA payloads, and the diagnostic components of each system are composed entirely of DNA to prevent its processing by ribonucleases, which would likely compromise its conditional function.

The conditional systems that follow display a continuous increase in design complexity, starting with a simple bimolecular switch that is able to detect a single input biomarker and release an ssRNA oligo when the RNA biomarker is present. From there, several pairs of cognate RNA/DNA hybrid constructs are characterized that perform conditional dsRNA release though differing diagnostic mechanisms. The first utilizes a diagnostic method whereby the two cognate constructs recognize neighboring sequence regions of a single input trigger to induce dsRNA release. The subsequent RNA/DNA hybrid pairs were all designed such that the diagnostic component responsible for the RNA biomarker that governed conditional function was completely contained within one of the two hybrids, while the cognate partner hybrid recognized a biomarker-dependent structural change of the first hybrid. Using this strategy, it is first demonstrated that dsRNA release can be induced by a single input, but then also that dsRNA release can be repressed following slight alterations to the design of the diagnostic component. Ultimately, a single cognate pair of RNA/DNA hybrid constructs are coupled that are able to detect multiple RNA triggers, with the release of dsRNA being dependent on the presence of one biomarker and the absence of a second. As a whole, the suite of conditional systems provides diversity in terms of the oligonucleotide output that can be generated, and the ability to either induce or repress oligonucleotide release based on the presence or absence of a specific RNA of interest.

### 3.2. A Beacon-Derived Conditional Switch Releases a Single-Stranded Oligonucleotide in the Presence of an RNA Trigger

**Figure 1:**
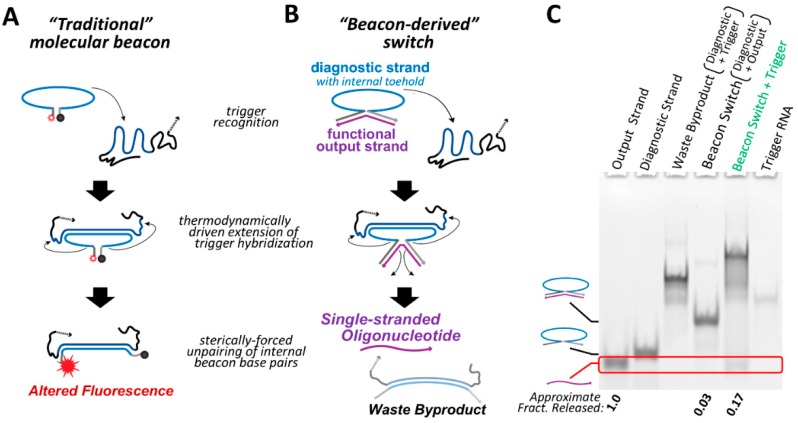
A conditional nucleic acid system based on the design of molecular beacons. (**A**) “Traditional” molecular beacons are fluorescence-based unimolecular diagnostic systems that adopt an initial hairpin structure. Hybridization of a trigger sequence complementary to the hairpin loop opens the hairpin and alters the fluorescence of the beacon by separating a fluorophore/quencher pair. (**B**) The “beacon-derived” switch is a biomolecular system composed of a diagnostic strand and an output strand. The output strand is hybridized to the 5′ and 3′ ends of the diagnostic strand creating a large bulge in the diagnostic strand. This bulge acts as an internal toehold. Hybridization of a trigger to this toehold region forms a persistent helix that outcompetes the internal pairing between the diagnostic and output strands, causing release of the output strand. (**C**) The conditional function of the beacon-derived switch was analyzed by 10% acrylamide non-denaturing PAGE and total staining with ethidium bromide. The beacon switch was assembled from the diagnostic and output strands. Addition of the trigger RNA to the pre-assembled beacon switch releases an output strand (red box) and shows generation of the expected waste byproduct. The fraction of output strand released was estimated by comparing the density of the output band to the output strand control lane of the same initial concentration. However, it should be noted that this approach is only semi-quantitative as it cannot be assumed that nucleic acid staining is completely uniform across the entirety of the gel. All samples were incubated for 30 min at 37 °C.

Traditional molecular beacons act as a unimolecular diagnostic tool, giving a fluorescent output signal that changes as a result of the presence of a specific oligonucleotide trigger [21] (Figure 1A). Rather than use fluorescence as an output signal, we have re-engineered the beacon system as a bimolecular switch construct that is able to release a single-stranded oligonucleotide upon recognition of a specific trigger sequence. Whereas traditional molecular beacons contain complementary regions at the 5′ and 3′ ends resulting in a hairpin structure (Figure 1A), the beacon switch is designed such that the output oligonucleotide is complementary across its length to the 5′ and 3′ ends of the diagnostic strand, generating a structure that resembles the shape of a horseshoe (Figure 1B). As with traditional molecular beacons, the diagnostic strand contains a large loop that is complementary to the trigger and serves as an internal toehold. Hybridization between the internal toehold and the trigger RNA acts as a thermodynamic driver that is intended to disrupt the pairing between the output strand and the diagnostic strand, resulting in the release of the single-stranded output.

Since the internal toehold of the diagnostic strand does not need to overlap with the 5′ and 3′ regions that are bound to the output oligonucleotide, essentially any set of trigger and target sequences can be implemented. The single-stranded output of the beacon switch could be composed of RNA or DNA depending on the desired function of the output strand. This conditional system could find application in instances where an irregular or diseased cellular state can be identified by a high copy number of a specific endogenous RNA, and the use of an AON, antagomir or other short single-stranded RNA would have a significant impact on rectifying the irregular state or inducing cell death.

For proof-of-principle illustration, a beacon switch was designed to respond to a fragment of the Kirsten rat sarcoma proto-oncogene (KRAS) mRNA as a trigger and release an RNA antagomir output strand in a conditional fashion. Analysis of beacon switch assembly and conditional output release was performed by non-denaturing polyacrylamide gel electrophoresis (PAGE) (Figure 1C). Assembly between the diagnostic strand and output strand to form the beacon switch is extremely efficient based on non-denaturing PAGE and total nucleic acid staining, with only trace amounts of single-stranded output strand observed after assembly. Co-incubation of the assembled beacon switch with the KRAS trigger at 37 °C results in the release of the output strand and the appearance of a band corresponding to the expected waste product. A higher migrating band also appears in this lane, which is presumed to be a trinary molecular complex of the assembled beacon switch bound to the trigger. The amount of output strand observed to be released is likely a lower limit of the amount of single-stranded oligo that is actually newly accessible following interaction with the trigger RNA. This is because only one end of the single-stranded output needs to be released by the diagnostic strand to allow complete hybridization with the trigger oligonucleotide (Figure S1). However, even a partially released single-stranded output oligo should be accessible to hybridize to a target RNA and still be able to perform its intended regulatory function.

### 3.3. Strand Exchange between RNA/DNA Hybrid Duplexes Can Be Facilitated by Toeholds That Target Adjacent Sequence Regions of an RNA Trigger

**Figure 2:**
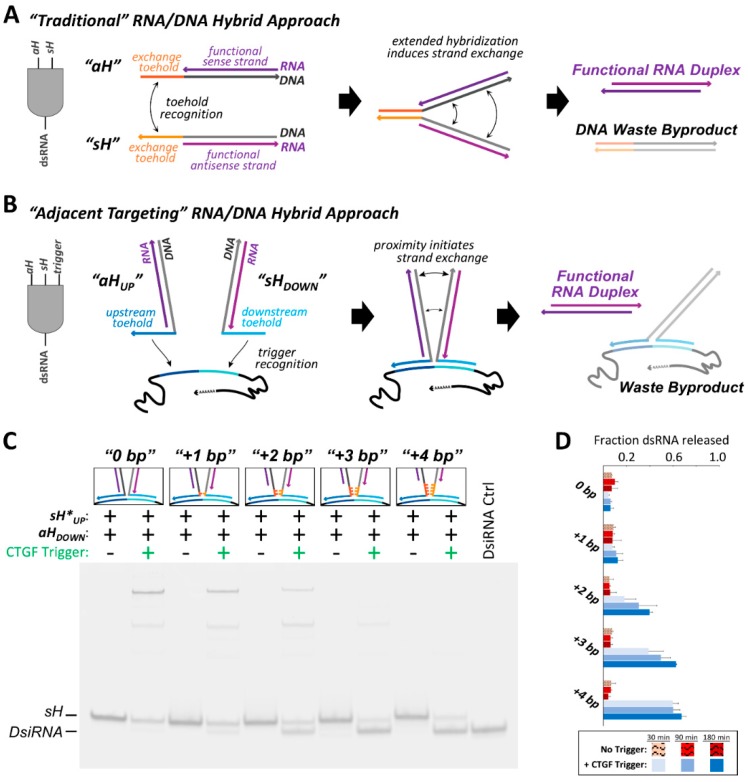
An RNA/DNA cognate pair system was designed to undergo conditional strand exchange by hybridizing to neighboring sites on an RNA trigger. (**A**) “Traditional” RNA/DNA hybrid pairs act as an 2-input *AND* gate. Hybridization between the single stranded toeholds of a sense hybrid (*sH*) and antisense hybrid (*aH*) initiates a thermodynamically driven strand exchange that generates a dsRNA duplex and DNA waste byproduct. (**B**) The “adjacent targeting” RNA/DNA hybrid system functions as a 3-input *AND* gate, requiring a hybrid pair as well as a specific RNA trigger sequence. The hybrid pair’s respective toeholds bind to regions of the trigger that are immediately upstream and downstream from one another. Anchoring the cognate hybrids in close proximity leads to initiation of the thermodynamically favorable strand exchange reaction and dsRNA release. (**C**) Five different cognate pairs of adjacent targeting hybrids were analyzed by 12% acrylamide non-denaturing PAGE for their ability to release a DsiRNA product. Each sense hybrid and the DsiRNA control assembly contained a 3′ 6-carboxyfluorescein (6-FAM) labeled sense RNA strand for visualization. The pairs of constructs differ in the number of DNA nucleotides inserted between the single-strand toehold and the RNA/DNA hybrid duplex. These inserted nucleotides were complementary between cognate hybrids, resulting in either 0, +1, +2, +3 or +4 DNA bp that can seed the strand exchange (colored orange). The presence or absence of each component is indicated above each lane. The samples in the gel depicted were all incubated for 180 min at 37 °C. (**D**) Analysis of the fraction of dsRNA released by hybrid pairs in the presence and absence of the RNA trigger following 30, 90 or 180 min incubations at 37 °C. Error bars indicate standard deviation of three replicate experiments. Indication of statistical significance between samples is reported in the supporting information.

Cognate RNA/DNA hybrid pairs were previously designed that harbor split functional RNAs, devised to release a recombined functional dsRNA through recognition of complementary single stranded toeholds (Figure 2A) [31,37,38]. The separated single strands composing the functional duplex can be referred to as the sense strand and the antisense strand, and each of these RNA strands were annealed to a complementary DNA oligo. These assembled RNA/DNA hybrids are denoted as the sense hybrid (*sH*) and the antisense hybrid (*aH*), respectively. The “traditional” approach to cognate hybrid design utilized complementary single stranded toeholds emanating from *sH* and *aH*, with hybridization of these toeholds to one another initiating RNA/DNA strand exchange. Here, we have redesigned the toeholds to be complementary to adjacent regions of an RNA trigger sequence, rather than complementary to one another. As the toeholds can no longer drive strand exchange by hybridization to one another, release of the dsRNA product is conditional on the presence of the RNA trigger molecule.

In this “adjacent targeting” incarnation of the RNA/DNA hybrid system, a fragment of the connective tissue growth factor (CTGF) mRNA was used as the RNA trigger sequence, acting as a template for DNA toehold binding which in turn initiates strand exchange (Figure 2B). Since the antisense hybrid binds upstream on the RNA trigger, it was termed *aH_UP_*. Similarly, the sense hybrid is referred to as *sH_DOWN_*. Binding of the cognate hybrid pair to the trigger RNA positions the two RNA/DNA hybrid regions adjacent to one another in space. The close proximity of the trigger-bound cognate hybrids will induce strand exchange through progressive hybridization of the trigger-bound DNA strands to one another, forming a three-way junction with the RNA trigger, and leading to formation and release of a dsRNA product. Like the beacon-derived switch, this activatable RNA/DNA hybrid system could find use in instances where a cell population of interest can be distinguished by the high relative expression level of an endogenous RNA. However, this RNA/DNA hybrid system (and those that follow) could be of use in cases where conditional generation of a double-stranded RNA is desirable, which could take the form of an RNA interference substrate, saRNA, aptamer, or another functionally relevant dsRNA. In this instance, the dsRNA product was designed as a 25/27-mer DsiRNA.

Formation of the dsRNA product was visualized by non-denaturing PAGE. The initial *aH_UP_*/*sH_DOWN_* cognate pair did not induce strand exchange and dsRNA release when co-incubated with the CTGF trigger for 180 min (Figure 2C, “0 bp”). In the presence of the RNA trigger, a large fraction of the hybrid constructs appear to be stuck in an intermediate complex displaying slow electrophoretic mobility. Presumably, this observed band corresponds to a state in which both RNA/DNA hybrids are bound to the trigger through their respective toeholds, but strand exchange in not stimulated. Despite no observed dsRNA release from this system, the strand exchange reaction is predicted to be thermodynamically favored (Figure S2). In an attempt to provide a greater driving force for strand exchange, additional sets of cognate hybrids pairs were designed in which additional complementary DNA nucleotides were inserted between the toehold region and the RNA/DNA hybrid region of each hybrid construct. These complementary nts were inserted to essentially serve as a nucleation site for strand exchange between the cognate partners once bound to the RNA trigger. In total, four additional hybrid pairs were designed which contained between 1 and 4 additional bps to seed the strand exchange (Figure 2C).

Increasing the number of complementary DNA bps inserted immediately prior to the RNA/DNA hybrid regions resulted in increased DsiRNA release (Figure 2C,D). Insertion of at least 2 DNA bps was needed to observe significant increases in DsiRNA release in the presence of the trigger RNA, as compared to background in the absence of the trigger after three hours (Table S1). Insertion of 3 bps appears to be enough to achieve close to the maximal degree of product duplex release, as increasing to 4 inserted bps results in negligible further increases in DsiRNA release after 180 min. However, the gel electrophoresis experiments suggest that insertion of additional bps does seem to speed up the rate at which this plateau of apparent maximal possible product release is reached, as the +4 bp pair releases significantly more dsRNA after 30 min than the +3 bp system, and likewise the +3 bp system shows greater release than the +2 bp hybrid pair (Figure 2D and Table S2). Despite the +3 bp and +4 bp hybrid pairs eventually reaching a similar level of dsRNA release after three hours, their differences in the fraction of dsRNA released at early time points suggests that the initiation of strand exchange within the adjacent targeting system may be impeded by slow kinetics. Interestingly, despite these systems containing complementary DNA nts that could potentially serve as toeholds to promote strand exchange in the absence of the trigger RNA, increasing the number of inserted seed base pairs up to four did not result in significant differences in the degree of non-triggered dsRNA release when co-incubated over the longest duration examined (Table 1 and Table S1).

### 3.4. A Responsive Structural Element Can Act to Conditionally Induce Strand Exchange between RNA/DNA Hybrids

**Figure 3:**
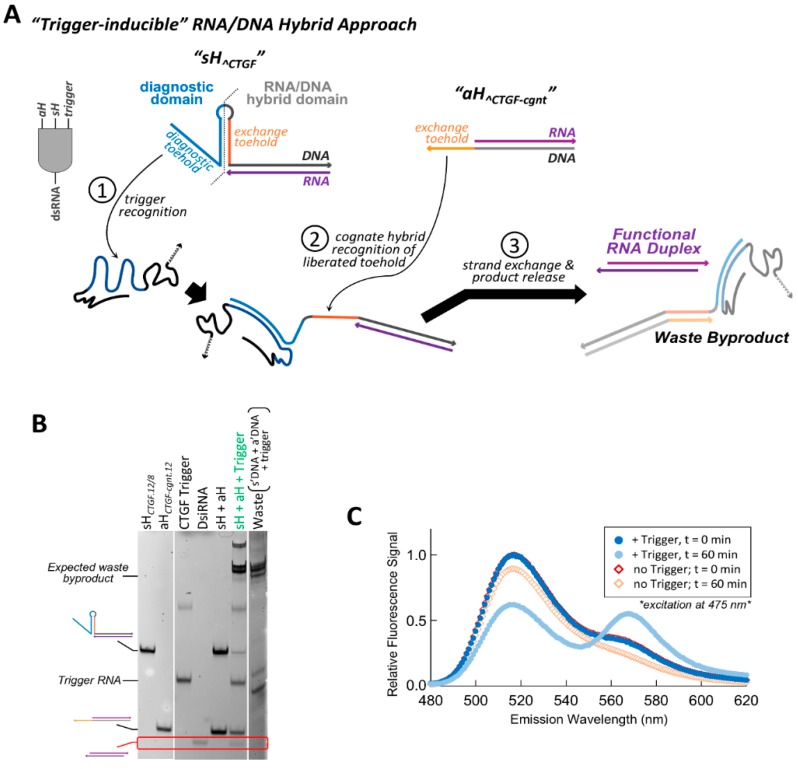
Incorporation of a structured responsive element can generate a trigger-inducible RNA/DNA hybrid system. (**A**) The inducible hybrid system functions as a three-input *AND* gate. The sense hybrid *sH_^CTGF.12/8_* contains a responsive DNA hairpin composed of a 12 bp stem and an 8 nt loop, and is flanked by an extended 5′ single strand that acts as a diagnostic toehold. Trigger hybridization to the diagnostic toehold progresses through the hairpin stem and unzips the hairpin (sequence regions colored blue). This liberates a previously sequestered toehold within *sH_^CTGF.12/8_* which can then hybridize with the complementary toehold of the cognate antisense hybrid, *aH_^CTGF-cgnt.12_*. Hybridization of these exchange toeholds (sequence regions colored orange) initiates strand exchange and releases a dsRNA product. (**B**) The function of this conditional system was assessed by 8% acrylamide non-denaturing PAGE and total staining with ethidium bromide. DsiRNA release is observed when the sense and antisense hybrids are co-incubated in the presence of trigger (red box). Formation of the expected waste product is observed by comparison to a control assembly of the s’ and a’ DNA strands with the trigger molecule. All samples were incubated for 30 min at 37 °C. (**C**) Förster resonance energy transfer (FRET) analysis was performed as another method to verify conditional dsRNA formation. *sH_^CTGF.12/8_* was assembled using a 3′ 6-carboxyfluorescein (6-FAM) (ex/em 495/520 nm) labeled sense RNA strand. *aH_^CTGF-cgnt.12_* was assembled using a 5′-AlexaFluor546 (ex/em 555/570 nm) labeled antisense RNA strand. The hybrids were mixed and incubated at 37 °C for one hour in the presence or absence of the RNA trigger. Fluorescence emission spectra were recorded at t = 0 and t = 60 min using excitation at 475 nm.

In an alternative approach for the implementation of conditional function within an RNA/DNA hybrid system, hybrid pairs were designed in which the accessibility of the toehold(s) needed to facilitate strand exchange was altered based on the presence or absence of a specific RNA trigger sequence. Although the adjacent targeting hybrid system described above performs its designed conditional function to release dsRNA, the fraction of dsRNA release for the best performing hybrid pair topped out at 0.67 after three hours. This second approach was pursued in an attempt to improve the efficiency of strand exchange and increase conditional dsRNA release. The “traditional” RNA/DNA hybrid methodology requiring the hybridization of complementary toeholds to one another for strand exchange serves as the basis of the conditional activation. We designate these single stranded toeholds as “exchange toeholds”, since they are required to initiate strand exchange. To create a hybrid system responsive to conditional activation, a structured hairpin element was incorporated in the DNA strand immediately adjacent to the RNA/DNA hybrid duplex region of the sense hybrid (Figure 3A). This DNA hairpin ultimately controls the reassembly fate of the split functional RNA. In its initial folded state, the DNA hairpin is designed to sequester the entire length of the exchange toehold sequence within its helical stem, preventing the toehold from readily interacting with the complementary exchange toehold of the cognate antisense hybrid. The resulting hybrid pair initially exists in an “off” state that is unable to initiate strand exchange.

A new single stranded toehold, termed the “diagnostic toehold”, is then implemented as a means to control the conditional activation of the hybrid by altering the accessibility of the exchange toehold imbedded within the DNA hairpin upon recognition of a specific RNA trigger sequence (Figure 3A). This single-stranded diagnostic toehold within the sense hybrid is positioned at the 5′ end of the DNA strand adjacent to the DNA hairpin (at the side opposite, the RNA/DNA hybrid region). By designing the sequence of the diagnostic toehold and the adjacent 5′ side of the DNA hairpin to be fully complementary to a region of an RNA trigger, hybridization of the trigger to the diagnostic toehold unzips the adjacent DNA hairpin and exposes the exchange toehold. Once the exchange toehold has been liberated, the complementary exchange toeholds of the hybrid pair can facilitate a strand exchange event and release a dsRNA output (Figure 3A). It is intended that this method of exchange toehold recognition, whereby the hybridization of complementary toeholds to one another forms a single duplex that can be directly extended by stacking additional DNA bps formed during RNA/DNA hybrid strand exchange, will exert a greater kinetic and/or thermodynamic drive than the three-way junction dependent method employed within the adjacent targeting system.

To illustrate the function of this “trigger-inducible” hybrid system, conditional hybrid constructs were designed to release a 25/27-mer DsiRNA when triggered by a fragment of the CTGF mRNA. The DNA strand of the sense hybrid was designed to contain a central hairpin with a 12 bp stem and 8 nt loop. This sense hybrid is referred to as *sH_^CTGF.12/8_*, as the hybrid is designed to stimulate dsRNA release in the presence of CTGF (“*^CTGF*”) and contains a DNA hairpin composed of a 12 bp stem and 8 nt loop (“*12/8*”). The exchange toehold within *sH_^CTGF.12/8_* is 12 nt in length and is initially completely sequestered within the DNA hairpin stem. The cognate partner hybrid is composed of an RNA/DNA hybrid duplex containing the DsiRNA antisense strand, with a 12 nt extension of the DNA strand at its 3′ end to encode the complementary exchange toehold. This hybrid is referred to as *aH_^CTGF-cgnt.12_* to reflect that it contains a 12 nt exchange toehold (“12”) and is the cognate partner (“cgnt”) to the CTGF-triggered *sH* hybrid (“*^CTGF*”).

Non-denaturing PAGE and total nucleic acid staining was used to examine interactions occurring between the cognate hybrids, as well as between the hybrids and the trigger RNA (Figure 3B). While not quantitative, initial analysis using a nucleic acid stain allowed for surveillance of all molecular species and products. As expected, no changes to the hybrids’ electrophoretic mobility is observed when incubated together at 37 °C in the absence of the trigger RNA, indicating that no interaction occurs between the hybrids and no dsRNA is released. Introduction of the RNA trigger activates *sH_^CTGF.12/8_* and induces the release of a dsRNA product when *aH_^CTGF-cognt12_* is also present. Higher migrating species are also observed when both hybrids are co-incubated with the trigger RNA. One of the high migrating bands corresponds to the expected waste product as indicated by similar migration of a control assembled from the RNA trigger and two DNA strands. An even slower migrating band is also observed and is likely to be a 5-molecule intermediate complex. Förster resonance energy transfer (FRET) experiments were performed to further verify the generation of the expected double stranded RNA product in the presence of the trigger molecule (Figure 3C). The cognate hybrids used for these FRET studies had a 3′ donor fluorophore on the RNA sense strand, and a 5′ acceptor fluorophore on the RNA antisense strand. In the absence of the RNA trigger, the FRET-labeled hybrid pair show no significant change in their emission spectrum after one hour at 37 °C. However, one hour after the introduction of the CTGF trigger a large decrease in donor emission (~515 nm) and increase in acceptor fluorescence (~565 nm) is observed, indicating formation of the DsiRNA duplex product.

### 3.5. Alteration of Structural Elements Was Explored as a Means to Optimize dsRNA Release from Cognate RNA/DNA Hybrids

**Figure 4:**
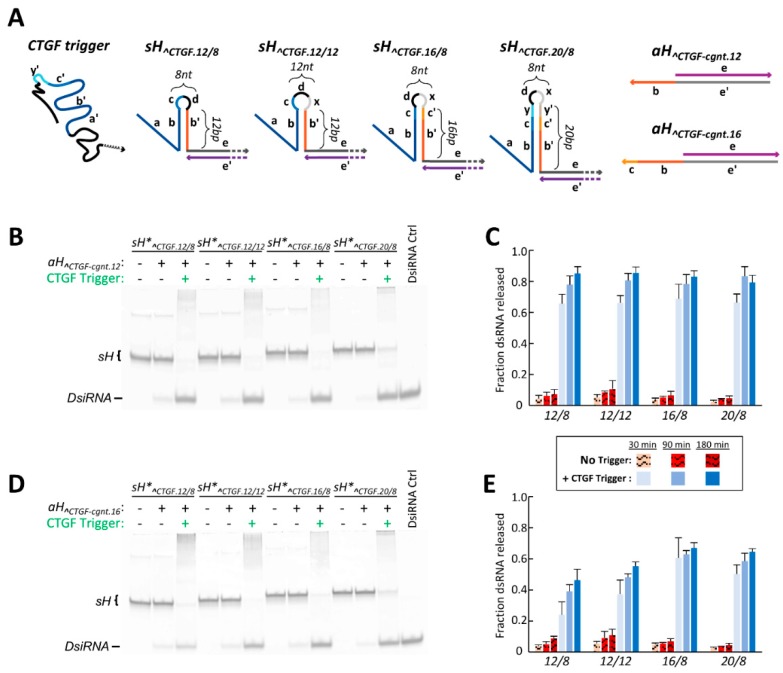
Effects of DNA structural alteration on the degree of trigger-inducible dsRNA release. (**A**) Four different sense hybrids that are responsive to the connective tissue growth factor (CTGF) trigger were designed, each having different features within the structured DNA hairpin. The hairpins differed in the size of their loop or the length of their stem. Two different cognate antisense hybrids were designed and differ in the length of their single-stranded toehold. Sequence regions are indicated by lowercase letters and different colors to convey sequence identity or sequence complementarity. (**B**,**D**) DsiRNA release in the presence and absence of trigger was assessed by 10% acrylamide non-denaturing PAGE for each sense hybrid paired with a cognate antisense hybrid exhibiting either (**B**) a 12 nt toehold (*aH_^CTGF-cgnt.12_*) or (**D**) a 16 nt toehold (*aH_^CTGF-cgnt.16_*). Each sense hybrid and the DsiRNA control contained a 3′ 6-carboxyfluorescein (6-FAM) labeled sense RNA strand for visualization and quantification. Gels in both (**B**) and (**D**) depict samples that were incubated for 30 min at 37 °C. (**C**,**E**) Analysis of the fraction of dsRNA released by the four sense hybrids paired with (**C**) *aH_^CTGF-cgnt.12_* or (**E**) *aH_^CTGF-cgnt.16_*, in the presence and absence of the RNA trigger following 30, 90, or 180 min incubations at 37 °C. Error bars indicate standard deviation of three replicate experiments. Indication of statistical significance between samples is reported in the supporting information.

Within the inducible hybrid system, the accessibility of one exchange toehold is impeded by being sequestered within a responsive DNA hairpin. This toehold becomes liberated upon opening of the hairpin in the presence of an RNA trigger and allows for a strand exchange to proceed. As such, altering the stability of this responsive hairpin structure, as well as the length and accessibility of the liberated exchange toehold once the hairpin is open, could potentially modulate the degree of strand exchange between a cognate hybrid pair. The initially characterized *sH_^CTGF.12/8_* hybrid contained a 12 bp DNA hairpin stem capped by an 8 nt loop. Three additional CTGF-triggered *sH* hybrids were designed to investigate how changing the structure of the responsive DNA hairpin affects strand exchange and dsRNA release (Figure 4A). The first of these variants maintains a 12 bp DNA hairpin stem but expands the hairpin loop from 8 to 12 nts. This hybrid is denoted sH*_^CTGF.12/12_*. The two additional *sH* variants maintain the original 8 nt hairpin loop, but contain hairpin stems of 16 and 20 bps in length. These hybrids are named *sH_^CTGF.16/8_* and *sH_^CTGF.20/8_*, respectively.

Each of the four *sH_^CTGF_* constructs were assembled with a fluorescently labeled RNA sense strand to quantitatively examine their ability to liberate a dsRNA duplex following strand exchange with *aH_^CTGF-cgnt.12_* in the presence and absence of the CTGF trigger RNA (Figure 4B). Interestingly, analysis using fluorescently labeled constructs revealed that the various *sH_^CTGF_* /*aH_^CTGF-cgnt.12_* hybrid pairs release a small fraction of dsRNA when incubated together in the absence of the trigger RNA. This was not originally observed in the initial qualitative experiments that utilized staining with ethidium bromide. The degree of non-triggered release among pairs of hybrid constructs was relatively minor after 30 min (~2–5% of signal) and was observed to marginally increase over time for each variant *sH_^CTGF_* construct paired with *aH_^CTGF-cgnt.12_* (Figure 4C). *sH_^CTGF.20/8_*, which is predicted to contain the most stable hairpin stem (Figure S3), exhibited the smallest degree of non-triggered DsiRNA release compared to other hybrids pairs after 30 min. Likewise, s*H_^CTGF.12/12_* was predicted to have the weakest hairpin structure and displayed the greatest extent of non-triggered DsiRNA release after 30 min. This trend persists at longer time points; however, differences in non-triggered DsiRNA release among variant hybrids pairs were not all statistically significant, especially at longer time points (Table S3).

Structural changes to the responsive DNA hairpin of the *sH_^CTGF_* hybrids resulted in negligible differences in trigger-induced dsRNA release between the four *sH_^CTGF_*/*aH_^CTGF-cgnt.12_* pairs assayed (Figure 4C). However, these constructs did show a 12–18% improvement in conditional dsRNA release over the best performing adjacent targeting hybrid pair after three-hour incubations with the CTGF trigger (Table 1). The lack of differences in triggered DsiRNA release among the variant *sH_^CTGF_* constructs was somewhat surprising based on the predicted change in free energy (ΔΔG) between the unbound and CTGF trigger-bound states for each hybrid’s responsive DNA element (Figure S3). However, it may be that the favorable change in free energy for each construct upon trigger binding is so great (ΔΔG < −25 kcal mol^−1^ for each) that the comparatively small differences in ΔΔG between the various *sH_^CTGF_* hybrids becomes inconsequential. Alternatively, differences in the ΔΔG of trigger binding could be offset by differences in steric accessibility of the newly liberated exchange toehold once the DNA hairpin has opened. Increasing the loop size or length of the hairpin stem increases the distance between the exchange toehold and the region bound by the RNA trigger once hybridized (Figure S3). This could in turn alter the accessibility of the liberated exchange toehold to the incoming cognate hybrid. The *sH_^CTGF.12/8_*/*aH_^CTGF-cgnt.12_* hybrid pair has the shortest nucleotide distance between the region bound by the trigger and its exchange toehold, and time course FRET experiments indicate the observed rate constant of dsRNA release is slower for this hybrid pairing than for any of the other three *sH_^CTGF_* hybrids paired with *aH_^CTGF-cgnt.12_* (Figure S4).

Extending the length of the exchange toehold was also explored as a means to boost triggered dsRNA release within the CTGF-inducible hybrid system. A variant *aH_^CTGF-cgnt_* hybrid was designed containing a 16 nt toehold and was termed *aH_^CTGF-cgnt.16_*. The toehold of *aH_^CTGF-cgnt.16_* was designed to encode the same 12 nt sequence as the *aH_^CTGF-cgnt._*_12_ toehold, with four additional nucleotides appended to the toehold’s distal end. These four additional nucleotides are complementary to corresponding regions within *sH_^CTGF.16/8_* and *sH_^CTGF.20/8_* and result in complete pairing of the 16 nt exchange toeholds between these cognate hybrids. However, these four added nucleotides at the distal end of the *aH_^_*_CTGF-cgnt.16_ toehold do not have complementary sequences in *sH_^CTGF.12/8_* and *sH_^CTGF.12/12_* (Figure 4A), leaving the distal end of the *aH_^CTGF-cgnt.16_* toehold unpaired.

Increasing the toehold length of the cognate antisense hybrid from 12 to 16 nucleotides was observed to have a negative impact on DsiRNA release when paired with any of the *sH_^CTGF_* variants (Figure 4D,E). The use of *aH_^CTGF-cgnt.16_* in place of *aH_^CTGF-cgnt.12_* had a negligible effect on the degree of non-triggered release but presented a large significant impediment to CTGF-triggered release in nearly all instances (Table S4). The extent of diminished triggered-release was most pronounced when *aH_^CTGF-cgnt.16_* was paired with *sH_^CTGF.12/8_* and *sH_^CTGF.12/12_*, suggesting that having non-complementary nucleotides at the distal end of the *aH_^CTGF-cgnt.16_* toehold interferes in some manner with the ability of the hybrids to promote the strand exchange reaction. As a way to compare the overall performance of the each conditionally-active hybrid pair, an “efficiency score” was determined for each time point examined. This efficiency score metric was calculated as the product of the fraction of triggered dsRNA release and the signal-to-noise ratio (triggered/non-triggered release). Larger scores indicated greater efficiency of conditional dsRNA release. Out of the eight pairs of CTGF-inducible hybrids and the five sets of adjacent-targeting hybrids, the *sH_^CTGF.20/8_*/*aH_^CTGF-cgnt.12_* pairing displays the highest efficiency score for each time interval that was examined (Table 1).

### 3.6. Redesigned Responsive Structural Elements Can Be Used to Inhibit Strand Exchange and Repress dsRNA Release

**Figure 5:**
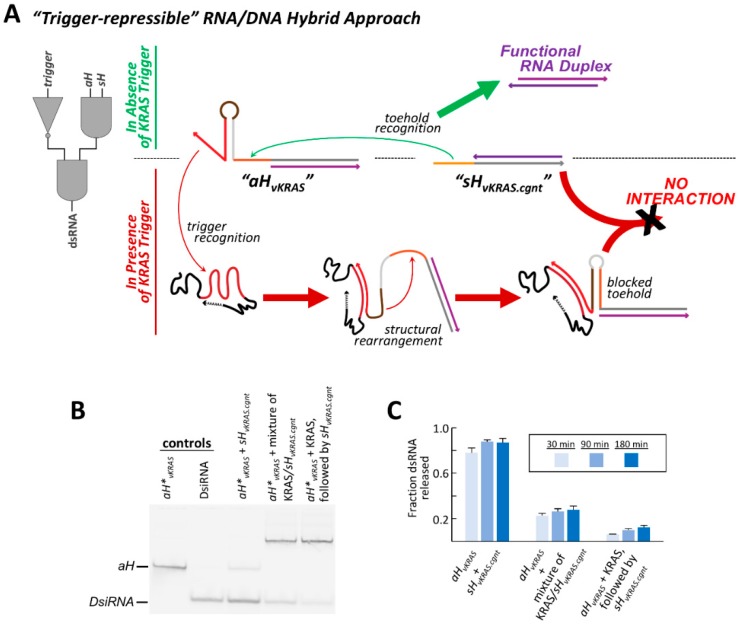
A redesign of the structured DNA responsive element allows for trigger-based conditional repression of dsRNA release. (**A**) A trigger-repressive hybrid system can be designed by combining a 2-input *AND* gate with a *NOT* gate. The antisense hybrid, *aH*∨*_KRAS_*, is designed to repress strand exchange in the presence of a trigger sequence derived from the Kirsten rat sarcoma proto-oncogene (KRAS) mRNA. If the trigger is absent, the exchange toehold of *aH*∨*_KRAS_* (sequence region colored orange) is freely accessible and can promote dsRNA release. If the trigger is present, its hybridization to the diagnostic toehold of *aH*∨*_KRAS_* (sequence region colored red) results in a structural rearrangement that blocks access to the exchange toehold and prevents interaction with the cognate sense hybrid, *sH*∨*_KRAS-cgnt_*. (**B**) The conditional function of this repressible system was assessed by 10% acrylamide non-denaturing PAGE. DsiRNA release from the *aH*∨*_KRAS_* /*sH*∨*_KRAS-cgnt_* pair was examined in three contexts: in the absence of the KRAS trigger (middle lane), when *sH*∨*_KRAS-cgnt_* and the KRAS trigger are premixed and added simultaneously to *aH*∨*_KRAS_* (2nd lane from right)*,* or when *aH*∨*_KRAS_* and the KRAS trigger are preincubated for 5 min prior to *sH*∨*_KRAS-cgnt_* addition (right lane). The KRAS trigger was added in 3-fold excess in both cases. The depicted gel shows samples incubated for 180 min at 37 °C once all components are present. (**C**) Analysis of the fraction of dsRNA released from the KRAS repressible system following 30, 90, or 180 min incubations at 37 °C. Error bars indicate standard deviation of three replicate experiments. Indication of statistical significance between samples is reported in the supporting information.

The concept and method of toehold sequestration used to impart conditional function within the trigger-inducible RNA/DNA hybrid system can be modified and redesigned to instead allow for the repression of strand exchange in the presence of a specific RNA trigger and thereby expands the degree of control over dsRNA release. In this embodiment, both exchange toeholds are initially free to undergo strand exchange, but one becomes sequestered into a DNA hairpin when interaction with an RNA trigger facilitates a structural rearrangement of that hybrid’s responsive structural element (Figure 5A). Such a system would be of interest in situations where a cellular state of interest cannot be identified by the high expression of a particular RNA, but rather by a significant under expression of a specific RNA relative to the normal population.

Whereas the previously described inducible hybrid pairs contain a responsive hairpin element within the DNA strand of *sH* that is triggered by CTGF, the repressible hybrid pair contains a responsive DNA element within *aH* that is responsive to the KRAS mRNA-derived trigger. This new hybrid is termed “*aH*∨*_KRAS_*” to indicate that dsRNA release from the hybrid is negatively impacted by the KRAS trigger. In the absence of the cognate RNA trigger, the most stable DNA fold of *aH*∨*_KRAS_* is that which results in a single stranded exchange toehold and a 14 bp DNA hairpin (Figure S5). When the trigger is present, however, it can bind to the 3′ diagnostic toehold present in *aH*∨*_KRAS_* and proceed to unzip the 14 bp hairpin, as the trigger is complementary to the entire 3′ side of the hairpin stem. As the initial 14 bp hairpin can no longer form, a structural rearrangement can occur where the exchange toehold pairs to the 12 nts that compose the apical loop of the original hairpin. This new hairpin structure makes the exchange toehold inaccessible to the cognate hybrid and represses the ability for the hybrid pair to release a dsRNA duplex (Figure 5A). 

The ability to repress hybrid strand exchange was examined for *aH*∨*_KRAS_* with its cognate hybrid, “*sH*∨*_KRAS-cgnt_*”, that contains a complementary 12 nt DNA exchange toehold extending from its RNA/DNA hybrid region. Analysis by non-denaturing PAGE at several time points illustrates that the cognate hybrids successfully undergo strand exchange and release dsRNA in the absence of the KRAS trigger (Figure 5B,C). However, when the KRAS trigger and *sH*∨*_KRAS-cgnt_* hybrid are premixed and added simultaneously to *aH*∨*_KRAS_*, DsiRNA release is repressed more than 3-fold compared to in the absence of KRAS. A second context was also examined, where *aH*∨*_KRAS_* was permitted to interact with the KRAS trigger for five minutes prior to the addition of the cognate *sH*∨*_KRAS-cgnt_* hybrid. This scenario allowed the responsive DNA hairpin to rearrange and adopt its alternative “off”-state structure before the cognate exchange toehold was present in the reaction mix. In this context, DsiRNA release is reduced 12-fold after 30 min at 37 °C compared to in the absence of trigger, and maintains more than 7-fold repression after 3 h (Figure 5C). FRET experiments further illustrate that strand exchange occurs quickly and efficiently in the absence of the KRAS trigger, but is severely impeded upon introduction of KRAS (Figure S6).

To illustrate that repression of dsRNA release is dependent on the presence of a trigger RNA with a specific nucleotide sequence, additional non-cognate trigger molecules were co-incubated with the repressive hybrid pair. Neither of the non-cognate trigger molecules tested resulted in a reduction in dsRNA release (Figure S7). This same degree of trigger specificity is observed for the CTGF-inducible hybrid system, as the *sH_^CTGF.20/8_*/*aH_^CTGF-cgnt.12_* hybrid pair are only observed to initiate dsRNA release in the presence of the CTGF trigger, and not when co-incubated with non-cognate trigger molecules (Figure S7). An orthogonal trigger-repressible system was also designed that is responsive to CTGF rather than KRAS, as a means to demonstrate versatility in accommodating various trigger sequence inputs, as well as an ability to position the response element at different locations within this generalized conditional system. In this system, the CTGF responsive DNA element was added to the sense hybrid rather than the antisense hybrid. Nonetheless, this cognate hybrid pair (*sH*∨*_CTGF_*/*aH*∨*_CTGF-cgnt_*) displays a repressed ability to generate dsRNA in the presence of the CTGF trigger, as intended (Figure S8).

### 3.7. Cognate Hybrids Pairs with Multiple Responsive Elements Allow for Multi-Trigger Regulation

Because the strand exchange reaction between cognate hybrid partners is dependent on the accessibility of a specific toehold sequence (exchange toehold) present on each of the two hybrids, it is possible to generate a system in which the accessibility of each toehold is under the control of a different RNA trigger sequence. In the case of the trigger-repressible hybrids, such as *aH*∨*_KRAS_*, the trigger RNA imparts no sequence constraints on the exchange toehold and allows the exchange toehold to be any sequence that permits proper folding. With this in mind, the exchange toehold of construct *aH*∨*_KRAS_* was designed to be complementary to the exchange toehold of the *sH_^CTGF_* hybrids characterized previously.

**Figure 6:**
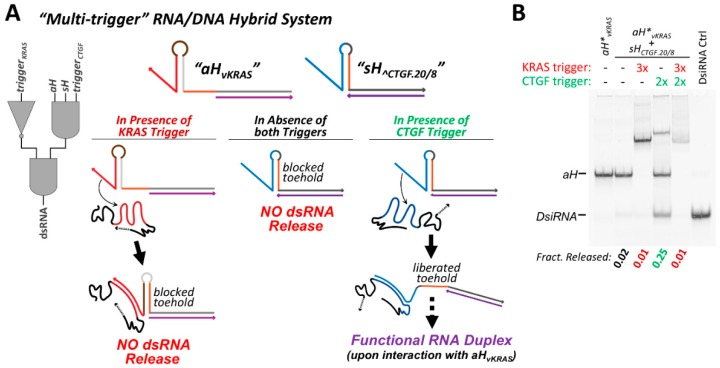
Multi-trigger systems can be composed in which each RNA/DNA hybrid contains a responsive DNA structural element. (**A**) A system comprising a 3-input *AND* gate and a *NOT* gate can be constructed by pairing *sH_^CTGF.20/8_* (activated by the connective tissue growth factor (CTGF) derived trigger) with *aH*∨*_KRAS_* (repressed by the Kirsten rat sarcoma proto-oncogene (KRAS) mRNA derived trigger). Co-incubation of the two hybrids results in no interaction. Both hybrids and the CTGF trigger are required for dsRNA release, while the presence of the KRAS trigger will inhibit strand exchange. (**B**) The multi-trigger system was assessed by 10% acrylamide non-denaturing PAGE. The fraction of DsiRNA released is indicated in the gel depicted, in the presence of indicated trigger combinations following 30 min incubation at 37 °C. The *sH* and *aH* hybrid were present at equimolar concentration, while the triggers were added at a 2-fold or 3-fold excess, as indicated. In samples when both triggers are present, they were added to premixed hybrids sequentially (KRAS followed by CTGF). The antisense hybrid and DsiRNA control in were assembled using a 5′-AlexaFluor546 labeled antisense RNA strand for the purpose of visualization and quantification.

Hybrid construct *sH_^CTGF.20/8_* was partnered with *aH*∨*_KRAS_* to generate a pair of conditional RNA/DNA hybrids whose function is dependent on the presence or absence of two RNA triggers, CTGF and KRAS (Figure 6A). The strand exchange reaction between these two hybrids is initially inhibited, as *sH_^CTGF.20/8_* initially exists in an “off” state and requires interaction with the CTGF trigger to promote strand exchange. *aH*∨*_KRAS_* is initially in an active state; however, the exchange toehold of *aH*∨*_KRAS_* becomes inaccessible upon interaction with the KRAS trigger. For efficient strand exchange to occur between this hybrid pair, the presence of the CTGF trigger is required, as well as the absence of the KRAS trigger.

The degree to which dsRNA could be conditionally released from this cognate hybrid pair was assessed by non-denaturing PAGE (Figure 6B) and FRET (Figure S9). In the absence of any trigger molecules, the *sH_^CTGF.20/8_*/*aH*∨*_KRAS_* hybrid pair releases very small amounts of dsRNA when co-incubated. The addition of the KRAS trigger to the hybrid pair reduces the degree of dsRNA release close to zero. However, if the CTGF trigger is added rather than the KRAS trigger, substantial release of dsRNA product occurs, as expected. Sequential addition of the KRAS trigger followed by the CTGF trigger to the hybrid pair results in very little dsRNA generation, suggesting that *aH*∨*_KRAS_* inactivation by the KRAS trigger occurs relatively quickly. Additional characterization was performed to examine how differences in the relative concentration of the two triggers affect dsRNA release. Various ratios of CTGF and KRAS trigger molecules were premixed and added to the co-incubating *sH_^CTGF.20/8_*/*aH*∨*_KRAS_* hybrid pair. As might be expected, increasing the relative amount of CTGF trigger (activating) to KRAS trigger (deactivating) increases the extent of dsRNA release (Figure S10). When equal amounts of the KRAS and CTGF triggers are added to the hybrid pair, the degree of dsRNA release is about 60% of the maximum amount of dsRNA released when an excess of CTGF trigger is added to the hybrids in the absence of the KRAS trigger. However, when the ratio of CTGF/KRAS triggers is varied away from 1:1, induction/repression of dsRNA release disproportionately favors the trigger that is present in a greater amount, beyond what would be predicted based on the trigger stoichiometry (i.e.,: when a 3:2 ratio of KRAS/CTGF is present, the fraction of dsRNA is less than 40% of the maximal dsRNA released in the absence of any KRAS).

### 3.8. Three-Strand RNA/DNA Hybrid Constructs Allow “Activated” Hybrids to Dissociate from Their Cognate Trigger

With both of the inducible and repressible conditional systems described above, the entirety of the hybrid construct containing the diagnostic toehold remains bound to the RNA trigger molecule following recognition and hybridization of the diagnostic toehold. However, one can imagine that there may be instances where the function of the conditional hybrid systems may benefit from allowing their RNA/DNA hybrid domains to freely diffuse away from their cognate trigger following hybridization through their diagnostic toehold/domain. A three-strand design approach was used to create an inducible hybrid that separates from the RNA trigger after hybridization. The design is based on that of the *sH_^CTGF.20/8_* hybrid. The 8 nt hairpin loop is removed, splitting the 20 bp hairpin into a duplex that assembles from two distinct DNA strands. One DNA strand retained the 5′ diagnostic toehold, while the other maintained the RNA/DNA hybrid region (Figure S11). This new three-strand hybrid was termed “*sH_^CTGF.20split_*” and works in conjunction with *aH_^CTGF-cgnt.12_*. Analysis by non-denaturing PAGE illustrates that the three-piece hybrid *sH_^CTGF.20split_* appears to function very similarly to that of *sH_^CTGF.20/8_*, although the three-piece hybrid seems to have a slight increase in its degree of non-triggered dsRNA release (Figure S11). 

A similar approach was used to investigate a three-strand repressible hybrid construct based on the *aH*∨*_KRAS_* hybrid. A nick was positioned within the 5′ strand of the DNA hairpin, aiming to maintain stable formation of the initial 14 bp hairpin and allow strand exchange in the absence of the KRAS trigger. Four different variants were designed to identify a nick position that retained the greatest conditional function. The function of the four variants partnered with *sH*∨*_KRAS-cgnt_* was examined by non-denaturing PAGE (Figure S12). Each of the three-strand repressible systems tested show a diminished ability to promote desirable dsRNA release in the absence of the KRAS trigger compared to the original design. This may stem from the possibility that a larger fraction of the three-strand hybrids initially adopt their “off” state when assembled. However, some three-strand systems did retain their repressible function. “*aH*∨*_KRAS.nick14_*”, where the nick was placed immediately below the hairpin loop and preserves the entire 14bp stem, displayed the greatest degree of conditional function. Progressively moving the nick down the stem resulted in continued loss of the responsive function to the KRAS trigger.

## 4. Discussion

Due to their ability to easily store and recognize information at the level of their primary sequence, nucleic acids serve as an excellent material for the construction of logic elements and performance of molecular computing [39,40]. Nucleic acid strand displacement and conformational change driven by single-stranded toehold interactions have found use in a wide variety of applications [41], with many great successes achieved by utilizing these techniques to perform complex diagnostics both in the test-tube [42,43,44] and in-cell [25,30,45]. However, the development of conditional therapeutics using similar logic-based diagnostic elements has lagged behind. In part, this is likely due to an increase in design constraints associated with development of therapeutics, as both the input and output oligonucleotides need to adhere to predetermined sequences. In the case of logic-driven diagnostic systems, the reporter output often induces translation of fluorescent proteins or the release of fluorescent nucleic acid probes, each of which are governed by structural changes within strands whose primary sequences are largely malleable to fulfill structural requirements. In most cases, these diagnostic systems function by the opening of a structure, or releasing a single-stranded oligonucleotide, both of which tend to be easier functions to design than the generation and release of a double-stranded duplex required in the case of RNAi-based applications. These increases in design complexity are evident in the conditional systems presented above. Conditional release of a single-stranded oligonucleotide that is triggered by a single input can be regulated by using a small, simple bimolecular system, whereas the release of duplex RNA similarly governed by a single input required much more complexity.

For a conditional RNA-based therapeutic system to be considered for practical application, it likely needs to fulfill three essential criteria: first, the RNA components must be protected from potential ribonuclease degradation; second, the sequences of the trigger input and the oligonucleotide output must be functionally and structurally independent from one another, such that either can be altered without necessitating a change in the other; and, third, the systems must be cheap and easy to produce, meaning that the use of modified nucleotides should be kept to a minimum. Previous incarnations of conditional RNA therapeutic systems have struggled to simultaneously fulfill these criteria. An early scheme for conditional Dicer substrate generation by Masu et al. utilized a hairpin design similar to that of a molecular beacon, where trigger binding to the loop unpairs an adjacent stem and allows formation of the functional sense-antisense hybrid [27]. This construct was successful at separating the trigger and target sequence elements, and demonstrated conditional gene silencing in HeLa cells. However, the system required co-delivery of a completely single-stranded cognate antisense strand leaving it vulnerable to ribonuclease degradation, while the hairpin construct required significant 2′-O-methyl (2′-OMe) modification to prevent Dicer from processing the initial hairpin stem. 

A similar approach was designed by Xie et al. whereby an antisense strand was pre-annealed to a chemically modified “protector” strand and this complex was co-delivered with a single-stranded sense RNA. The protector strand contained significant overhangs on each end and was completely complementary to a trigger RNA, allowing hybridization to the trigger to drive antisense release and siRNA formation [28]. This approach though fails to decouple trigger and output sequences, while suffering from the same ribonuclease susceptibility and extensive modification issues of the hairpin based approach. Hochrein et al. have constructed some of the most promising systems conceptually for the generation of a Dicer substrate siRNA. Their system utilizing stable small conditional RNAs both effectively separates trigger and output sequence requirements and provides nuclease protection of functional RNA regions through hybridization to 2′-OMe RNA [29], but again the significant use of chemically modified nucleotides can hinder efficient and cost effective synthesis. This use of extensive 2′-modification to not only protect regions of functional RNA, but also to prevent off-site Dicer cleavage is a reoccurring theme among these previous generations of conditional systems. Work by Kumar et al. explored a completely different approach by genetically expressing a pri-miRNA-like conditional construct from a plasmid [26]. While the system was able to perform conditional silencing in cell culture, this system required the delivery of modified oligonucleotide triggers to induce activation and processing by Drosha. Additionally, the need to prepare and express constructs from a plasmid brings additional complications.

The original incarnation of split-function RNA/DNA hybrid pairs from Afonin et al. lacks a true diagnostic component that would be able to sense an RNA biomarker but was able to successfully separate the output oligonucleotide sequences from the toehold regions that controlled the conditional strand–exchange reaction. Importantly, they were able to demonstrate that formation of an RNA/DNA hybrid duplex both protected the RNA from degradation and prevented the initial duplexes from being processed by Dicer [31]. As described above, these “traditional” RNA/DNA hybrid duplexes served as a jumping off point for the development of a new generation of conditional RNA-based therapeutics, by inserting or appending new diagnostic components able to respond to the presence of RNA biomarkers. These new systems fulfill our outlined criteria for conditional therapeutics by combining the nuclease resistance of the RNA/DNA hybrid technology with independent diagnostic components. In addition, the earlier generations of conditional systems described above each functioned as Boolean *AND* gates, releasing a double stranded RNAi substrate based on the presence of a single RNA trigger. We demonstrate in this current work that these next-generation RNA/DNA hybrid systems can be based on *AND* gates, *NOT* gates and combinatorial systems able to sense multiple biomarker triggers. Furthermore, although the proof of principle demonstrations we have presented here release a single dsRNA duplex from each RNA/DNA hybrid system, it should be possible to release multiple different dsRNA products in a conditional fashion from a single pair of conditional RNA/DNA hybrids, as this has been demonstrated previously with the “traditional” hybrid approach [37]. As an assembled collection, this suite of nucleic acid logic systems represents a robust toolkit for the conditional generation of nucleic acid species that encode a specific sequence and function while serving as a foundation for future therapeutic development.

## Figures and Tables

**Table 1:** Summary of observed dsRNA release from connective tissue growth factor (CTGF) trigger-inducible hybrid pairs. The average fraction of dsRNA release is reported in the presence and absence of CTGF trigger, at each of three time intervals examined. An efficiency score metric is determined for each hybrid pair at a given time point, with a larger score indicating better efficiency of conditional dsRNA release. The efficacy score takes into account both the fraction of dsRNA released and the signal-to-noise ratio. It is calculated as (fraction of triggered release) * (fraction triggered release/fraction non-triggered release). The hybrid pairing that yields greatest efficiency score at each of the three time intervals examined is bolded.

Hybrid Pair		Fraction dsRNA Released, 30 min			Fraction dsRNA Released, 90 min			Fraction dsRNA Released, 180 min		
Sense Hybrid	Antisense Hybrid	Non-Triggered	CTGF-Triggered	Efficiency Score	Non-Triggered	CTGF-Triggered	Efficiency Score	Non-Triggered	CTGF-Triggered	Efficiency Score
*sH_DOWN.0bp_*	*aH_UP.0bp_*	0.07 ± 0.004	0.04 ± 0.01	0.03	0.10 ± 0.03	0.06 ± 0.01	0.04	0.07 ± 0.05	0.06 ± 0.03	0.05
*sH_DOWN+1bp_*	*aH_UP+1bp_*	0.09 ± 0.02	0.08 ± 0.02	0.07	0.08 ± 0.02	0.11 ± 0.06	0.15	0.08 ± 0.08	0.12 ± 0.04	0.20
*sH_DOWN+2bp_*	*aH_UP+2bp_*	0.05 ± 0.03	0.18 ± 0.10	0.62	0.05 ± 0.01	0.31 ± 0.16	1.79	0.06 ± 0.05	0.40 ± 0.03	2.69
*sH_DOWN+3bp_*	*aH_UP+3bp_*	0.08 ± 0.01	0.39 ± 0.13	2.01	0.06 ± 0.02	0.49 ± 0.08	4.00	0.06 ± 0.02	0.63 ± 0.01	6.29
*sH_DOWN+4bp_*	*aH_UP+4bp_*	0.07 ± 0.04	0.60 ± 0.05	5.28	0.06 ± 0.01	0.60 ± 0.06	5.63	0.04 ± 0.02	0.67 ± 0.04	10.77
*sH_^CTGF12/8_*	*aH_^CTGF-cgnt.12_*	0.05 ± 0.02	0.66 ± 0.06	9.5	0.06 ± 0.02	0.78 ± 0.06	9.7	0.07 ± 0.03	0.85 ± 0.04	10.0
*sH_^CTGF.12/12_*	*aH_^CTGF-cgnt.12_*	0.05 ± 0.02	0.66 ± 0.05	8.1	0.09 ± 0.01	0.81 ± 0.05	7.6	0.11 ± 0.05	0.85 ± 0.04	6.8
*sH_^CTGF.16/8_*	*aH_^CTGF-cgnt.12_*	0.04 ± 0.01	0.69 ± 0.09	13.3	0.05 ± 0.01	0.78 ± 0.06	12.0	0.07 ± 0.02	0.83 ± 0.04	10.5
*sH_^CTGF.20/8_*	*aH_^CTGF-cgnt.12_*	**0.02 ± 0.01**	**0.66 ± 0.06**	**17.8**	**0.04 ± 0.01**	**0.83 ± 0.06**	**17.5**	**0.05 ± 0.02**	**0.79 ± 0.05**	**13.6**
*sH_^CTGF12/8_*	*aH_^CTGF-cgnt.16_*	0.04 ± 0.01	0.24 ± 0.08	1.5	0.05 ± 0.02	0.39 ± 0.04	3.0	0.09 ± 0.01	0.46 ± 0.07	2.4
*sH_^CTGF.12/12_*	*aH_^CTGF-cgnt.16_*	0.06 ± 0.01	0.37 ± 0.09	2.5	0.09 ± 0.04	0.48 ± 0.02	2.6	0.11 ± 0.04	0.55 ± 0.03	2.8
*sH_^CTGF.16/8_*	*aH_^CTGF-cgnt.16_*	0.05 ± 0.01	0.61 ± 0.13	7.5	0.05 ± 0.01	0.63 ± 0.02	7.2	0.07 ± 0.02	0.67 ± 0.03	6.5
*sH_^CTGF.20/8_*	*aH_^CTGF-cgnt.16_*	0.03 ± 0.01	0.50 ± 0.06	9.4	0.04 ± 0.004	0.59 ± 0.05	9.7	0.05 ± 0.01	0.64 ± 0.02	9.2

## Supplementary Materials

The following are available online at https://www.mdpi.com/2079-4991/9/4/615/s1: a detailed description of conditional RNA/DNA hybrid construct design, all RNA and DNA sequences used in this study, Figure S1: free energy calculations pertaining to the beacon-derived switch, Figure S2: Free energy calculations pertaining to the +0 bp adjacent targeting hybrid system, Figure S3: Free energy calculations pertaining to the responsive DNA hairpin elements of the *sH_^CTGF_* hybrids, Figure S4: Time course FRET experiments of variant *sH_^CTGF_* hybrids paired with *aH_^CTGF.cgnt12_*, Figure S5: Free energy calculations pertaining to the responsive DNA hairpin element of *aH*∨*_KRAS_*, Figure S6: Time course FRET experiments of *aH*∨*_KRAS_* paired with *sH*∨*_KRAS.cgnt_*, Figure S7: PAGE analysis of conditional dsRNA in presence of non-cognate trigger molecules, Figure S8: PAGE analysis of a CTGF-repressible conditional hybrid pair, Figure S9: Time course FRET experiments of a multi-input conditional hybrid pair, Figure S10: Effect of trigger titration on dsRNA release from a multi-input conditional hybrid pair, Figure S11: PAGE analysis of a 3-piece trigger-inducible RNA/DNA hybrid, Figure S12: PAGE analysis of a 3-piece trigger-repressible RNA/DNA hybrid, Table S1: Statistical significance of dsRNA release from a single adjacent-targeting hybrid pair at various timepoints, Table S2: Statistical significance of dsRNA release between various adjacent-targeting hybrid pairs at a single time point, Table S3: Statistical significance of dsRNA release among various trigger-inducible *sH_^CTGF_* hybrids pairs at a single time point, Table S4: Statistical significance of dsRNA released for a given *sH_^CTGF_* when paired with *aH_^CTGF-cgnt.12_* versus *aH_^CTGF-cgnt.16_*, Table S5: Statistical significance of dsRNA release from a single CTGF trigger-inducible hybrid pair at various timepoints, Table S6: Statistical significance of dsRNA released for the KRAS-repressible hybrid pair.
